# Supplementary material for: Isolation of Candida Species Is Associated with Comorbidities, Prolonged Mechanical Ventilation, and Treatment Outcomes in Surgical ICU Patients, a Cross-Sectional Study
Source: J Fungi (Basel). 2024 Oct 28;10(11):743. doi: 10.3390/jof10110743 (PMC11595781; doi:10.3390/jof10110743)
Supplement: Supplementary file 1 [file jof-10-00743-s001.zip › jof-3207357-supplementary.pdf]

Journal of *Fungi*

Article

Isolation of *Candida* Species Is Associated with Comorbidities, Prolonged Mechanical Ventilation, and Treatment Outcomes in Surgical ICU Patients, a Cross-Sectional Study

Josipa Glavaš Tahtler [1,2,*], Ana Cicvarić [1,2], Despoina Koulenti [3,4], Marios Karvouniaris [5], Maja Bogdan [2,6], Kristina Kralik [2], Irena Krajina Kmoniček [1,2], Marina Grbić Mlinarević [1] and Slavica Kvolik [1,2,*]

Table S1. Azole resistance in *Candida* species in the surgical patients admitted into ICU from May 2016 to June 2023.

		Candida species	N	%
C. albicans			426	73
Candidae non-albicans	Fluconazole resistant	*C. glabrata*	53	9.1
		C. krusei	4	0.7
		C. guilliermond	2	0.3
	Fluconazole sensitive	*C. parapsilosis*	45	7.7
		C. tropicalis	13	2.2
		C. kefyr	7	1.2
		C. rugosa	4	0.7
		C. famata	4	0.7
		C. lusitaniae	4	0.7
		C. metapsilosis	1	0.2
		C. orthopsilosis	1	0.2

Table S2. A comparison of comorbidities in surgical patients with Candida isolation during the ICU treatment, and in the control group of surgical ICU patients.

Comorbidities	*Candida* (N=236)	Control (N=261)	Total	*P**
**Heart diseases**	**122 (52)**	96 (37)	218 (44)	**0.001**
**Vascular**	138 (58)	172 (66)	310 (62)	0.09
**Respiratory**	**137 (58)**	105 (40)	242 (49)	**<0.001**
**Coagulopathy**	**40 (17)**	15 (6)	55 (11)	**<0.001**
**Polytrauma**	33 (14)	39 (15)	72 (14)	0.76
**Neurological**	102 (43)	90 (34)	192 (39)	0.05
**Renal**	**79 (33)**	54 (21)	133 (27)	**0.001**
**Urologic**	50 (21)	42 (16)	92 (19)	0.14
**Gastrointestinal**	130 (55)	133 (51)	263 (53)	0.36
**Hepatobiliary**	**54 (23)**	37 (14)	91 (18)	**0.04**
**Sepsis**	**94 (40)**	41 (16)	135 (27)	**<0.001**
**Neoplasm**	62 (26)	131 (50)	193 (39)	**<0.001**
**Gyn-obstetric**	8 (3)	6 (2)	14 (3)	0.46
**Metabolic**	87 (37)	47 (18)	134 (27)	**<0.001**
**Endocrinopathies**	54 (23)	73 (28)	127 (26)	0.27
**Psychiatric**	48 (20)	47 (18)	95 (19)	0.52
**Soft tissue infections**	**44 (19)**	12 (5)	56 (11)	**<0.001**

For each comorbidity, a number (%) of patients with specified diseases was shown. *χ^2 test, statistically significant differences are bolded.

Table S3. Type of surgery and presence of bacterial infection during the ICU stay.

	Candida (n=236)	Controll (n=261)	Total (n=598)	*P**
**Type of surgery (n = 448)**	210 (89)	238 (91)	448 (90)	0.61
**Craniotomy**	44 (21)	43 (18)	87 (19)	**0.04**
**Laparotomy**	116 (56)	116 (49)	232 (52)	
**Thoracotomy**	6 (3)	18 (8)	24 (5)	
**Trauma, extremities**	11 (5)	8 (3)	19 (4)	
**2 systems**	11 (5)	10 (4)	21 (5)	
**Other**	22 (10)	43 (18)	65 (15)	
**Reoperation**	118 (51)	41 (16)	159 (32)	**<0.001**
**Bacterial infections†**				
**No**	51 (22)	52 (40)	103 (28)	
**Community-acquired**	72 (31)	23 (18)	95 (26)	**<0.001**
**Hospital-acquired**	113 (48)	54 (42)	167 (46)	**<0.001**

A number (%) of patients with specified conditions is shown. Some surgical patients, i.e. with poly-trauma or brain injury, were not operated on during the ICU stay. *χ^2 test, statistically significant differences are bolded. †Only patients from whom samples were taken for microbiological analysis are presented. Microbiological samples were not taken from all patients in the control group, but only from those with signs of infection.

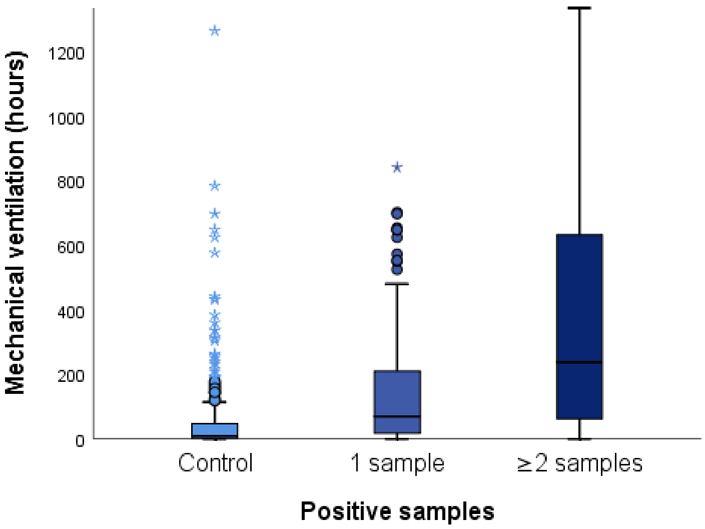

Figure S1. Mechanical ventilation in the surgical intensive care unit (ICU) patients according to the number of positive *Candida* species. Boxplots present medians and interquartile ranges of mechani-cal ventilation in a control group of ICU patients (N=261) and in patients with *Candida* isolation in one sample (N=137) and ≥2 samples (N=98) during their ICU stay.

Table S4. Prediction of the probability of a negative outcome (death) in the multivar-iate logistic regression in the whole population.

Multivariate regression*	ß	P	OR	95% CI
Sepsis	0.819	**0.04**	2.27	1.03 - 5.03
Constant	-6.57	**<0.001**		

*adjusted for: patient's age, SAPS II score, days of ICU, type of admission classified as elective/non-elective.

Table S5. Prediction of the probability of a negative outcome (death) in the multivariate logistic regression in the patients with *Candida* isolation.

Multivariate regression*	ß	P	OR	95% CI
Sepsis	0.974	**0.04**	2.65	1.02 do 6.88
Constant	-6.07	**<0.001**		

* adjusted for: age, SAPS II score, days of ICU, type of admission classified as elective/non-elective.
